# Comparison of Gut Microbiota of Yaks From Different Geographical Regions

**DOI:** 10.3389/fmicb.2021.666940

**Published:** 2021-06-07

**Authors:** Wenwen Liu, Qiang Wang, Jiajia Song, Jinwei Xin, Songshan Zhang, Yuanhua Lei, Yuanli Yang, Peng Xie, Huayi Suo

**Affiliations:** ^1^Institute of Animal Sciences, Chinese Academy of Agricultural Sciences, Beijing, China; ^2^College of Food Science, Southwest University, Chongqing, China; ^3^State Key Laboratory of Hulless Barley and Yak Germplasm Resources and Genetic Improvement, Lhasa, China; ^4^Institute of Animal Science and Veterinary, Tibet Academy of Agricultural and Animal Husbandry Sciences, Lhasa, China

**Keywords:** high-throughput sequencing, gut microbiota, yak, diversity, geographical regions

## Abstract

Gut microbiota are closely linked to host health and adaptability to different geographical environments. However, information on the influence of different geographical conditions on the intestinal microbiota of yaks is limited. In this study, 18 yak fecal samples were collected from three regions of China, namely Shangri-la, Lhasa, and Yushu, and were analyzed via high-throughput sequencing. The alpha diversity, as measured by the Shannon, ACE, and Chao indices, was the highest in the Shangri-la samples. Principal coordinate analysis detected significant differences in the composition of the intestinal microbiota of yaks from different regions. A total of six phyla, 21 families, and 29 genera were identified in the fecal samples. The dominant phyla in the samples were Firmicutes and Bacteroidetes, and the most abundant family was Ruminococcaceae. In addition, *Ruminococcaceae_UCG-005* was the predominant genus and was more abundant in Yushu samples than in other samples. However, the predicted functional gene composition of the gut microbiota of yaks from different regions was similar. Our results revealed that geographical conditions influence the diversity and composition of the intestinal microbiota of yaks.

## Introduction

Yak (*Bos grunniens*) is a representative indigenous ruminant in the Qinghai-Tibetan Plateau, which is well adapted to live in environments of severe cold, poor foraging resources, high ultraviolet radiation, and low oxygen levels (Huang et al., 2012). Most yaks are found in South-Central Asia (China, Mongolia, Russia, and other countries) (Li et al., 2018). China houses approximately 14 million yaks belonging to 12 yak breeds, and most yaks are distributed in Qinghai, Yunnan, Tibet, and Gansu provinces (He et al., 2011; Wu, 2016). This population represents approximately 95% of the world’s yak population (Zhu et al., 2018). Yaks also play vital roles in Qinghai-Tibetan Plateau agriculture as they provide milk, fur, meat, and transport for local herdsmen (Wiener et al., 2003). Yaks are associated with many harsh environmental conditions, such as low oxygen levels, low temperatures, and high altitudes, which limit the quality and availability of food (Wang et al., 2011; Zhou et al., 2017).

The environmental conditions in which yaks live vary greatly from region to region. For instance, Shangri-la is located in the northwest of Yunnan Province and the hinterland of the Qinghai-Tibet Plateau Hengduan mountain area and belongs to the temperate climate and plateau climate zone. The average annual temperature of the Shangri-la plateau is approximately 5°C (Duan et al., 2019). Lhasa (Tibet) is located in the central and southern parts of the Qinghai-Tibet Plateau and has an alpine climate. The temperature here is markedly different between the day and nigh, and the day is long. Yaks also live in regions with hypoxia and sparse vegetation (Jia et al., 2018). Yushu (Qinghai Province) is located in the eastern part of the Qinghai-Tibet Plateau, where yaks live in areas with low oxygen partial pressure, large temperature differences between the day and night, intense radiation, and short grass growing period (Qin et al., 2019). In different regions, food availability is different for yaks. The diverse yak food include native grass, manually planted forage, and artificial forage supply. Hence, yaks have unique gut microbiota, which enables them to stay healthy and survive in harsh environments. Previous studies have shown that trillions of microbial cells, termed microbiota, living in the gastrointestinal tract (GIT), play an important role in host adaptation (Xin et al., 2019). Gastrointestinal microbiota are essential for immune response, GIT development, nutrient absorption, and metabolism in ruminants (Morgavi et al., 2015). Moreover, recent research has revealed that the complex microbiota of the rumen, particularly bacteria in the rumen, play a vital role in nutrient metabolism by providing energy and essential nutrients and converting plant fibers and proteins into microbial proteins and volatile fatty acids for the host (Wu et al., 2016). Microbial communities in mice have been found to modulate intestinal angiogenesis and bone mass density (Reinhardt et al., 2012; Sjögren et al., 2012). Furthermore, studies in mice have suggested that metabolic disorders are strongly linked to the composition of gut microbiota (Malmuthuge and Guan, 2016). For example, research on obesity has revealed that Rikenellaceae and Ruminococcaceae were more abundant in mice fed a high-fat diet, which is related to ingested diet, type 2 diabetes, and obesity (Kim et al., 2012). Thus, determining the diversity in gut microbiota of yaks will provide insights into their health and development.

High-throughput sequencing (HTS) technology is a general term applied to new genomic sequencing technologies, such as Illumina MiSeq, which can be done more inexpensively and faster than traditional approaches, making HTS a powerful tool for assessing microbial diversity (Pallen et al., 2010; Zhu et al., 2018). For instance, Zhou et al. (2016) used HTS technology to analyze microbial communities in the cecal tubes in Tibetan chickens and found that the gastrointestinal microbiota of Tibetan chickens from six different geographical environments diverged slightly. Zhao et al. (2018) found significant differences in the composition of the intestinal microbiota of Chinese rhesus macaques from six different geographical environments using MiSeq technology. In a study on the gut microbiota of obese individuals, Angelakis et al. (2019) used high-throughput 16S rRNA gene sequencing to reveal that there were significant differences in the intestinal microbiota of individuals having different geographical origins. Yang et al. (2020) used Illumina MiSeq to study the composition of *Bifidobacterium* communities and gastrointestinal microbiota of various individuals and found significant differences in the composition of microbial genera in samples grouped by region and age. Recent research in yaks demonstrated that the diversity of intestinal microbiota is significantly different in various parts of the rumen and varies with different feeding methods (Ren et al., 2020; Zhang X.-L. et al., 2020). However, to our knowledge, no research has been conducted to understand the effects of different geographical conditions on yak gut microbiota.

Considering the importance of the gut microbiome, this study compared the composition of microbiota of yaks from different regions. We characterized the microbial diversity of yaks from different regions and predicted microbial functions based on gene composition using HTS technology. The results of this study not only demonstrate that the gut microbiota of yaks are significantly affected by different geographical regions but also improve our understanding of the species composition of gut microbiota under different geographical conditions. Moreover, this study is the first to report a difference in the gut microbiota of yaks from different geographical regions, encountering different conditions.

## Materials and Methods

### Animals and Fecal Sample Collection

**FIGURE 1:**
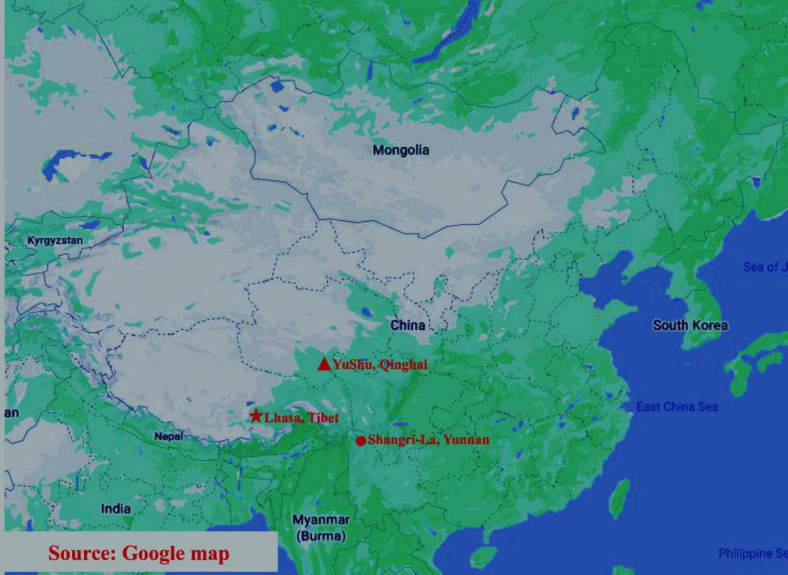
Sampling sites in this research, including Lhasa, Yushu, and Shangri-la. The three sampling locations are represented by different shapes on the map. Triangle: Yushu (Qinghai Province); pentagon: Lhasa (Tibet Autonomous Region); circle: Shangri-La (Yunnan Province).

Eighteen adult male yaks were sampled from three different geographical regions of China, namely Lhasa (Tibet Autonomous Region), Yushu (Qinghai Province), and Shangri-la (Yunnan Province). For each region, stool samples were collected from six yaks from a single group that exclusively grazed on natural pastures in the summer. After each yak defecated, the fresh fecal sample was immediately transferred into sterile tubes using sterile gloves and spoons. The tubes were then frozen in liquid nitrogen and transported to the laboratory on dry ice. The samples were stored at −80°C until further analysis. The sampling sites of this study are shown in Figure 1.

### DNA Extraction, PCR Amplification, and Sequencing

Microbial genomic DNA was extracted from fecal samples using a FastDNA^TM^ Fecal Kit (MP Biomedicals, Santa Ana, CA, United States) according to the manufacturer’s protocol (Shanks et al., 2011). The V3–V4 hypervariable regions of the bacterial 16S rRNA genes were PCR-amplified using the forward primer 338F (5′-ACTCCTACGGGAGGCAGCAG-3′) and reverse primer 806R (5′-GGACTACHVGGGTWTCTAAT-3′). PCR reactions contained 4 μL of 5 × FastPfu Buffer, 2 μL of 2.5 mM dNTPs, 0.8 μL of each primer (5 μM), 0.4 μL of FastPfu polymerase, and 10 ng of template DNA in a volume of 20 μL. Cycling parameters included initial denaturation at 95°C for 3 min, followed by 20 cycles of denaturation at 95°C for 30 s, annealing at 55°C for 30 s, and elongation at 72°C for 30 s, and final elongation for 10 min at 72°C. PCR products were recovered by electrophoresis using 2% agarose gels. Subsequently, the recovered PCR products were purified using an AxyPrep DNA Gel Extraction Kit (Axygen Biosciences, Union City, CA, United States) and eluted with Tris–HCl. The concentration of the purified PCR products was checked using electrophoresis in 2% agarose gels and quantified using QuantiFluor^TM^-ST (Promega, Madison, WI, United States). Illumina paired-end sequencing libraries were prepared using the purified PCR products according to the standard sample preparation protocol for the Illumina MiSeq platform (Illumina, San Diego, CA, United States). Thereafter, the libraries were sequenced using the Illumina MiSeq PE300 platform, and 2 × 300 bp PE reads were generated.

### Data and Statistical Analysis

Clean reads were obtained by filtering the raw sequences using Trimmomatic. Paired-end reads were merged using Flash (V1.2.11). These sequences were grouped into operational taxonomic units (OTUs) based on 97% sequence identity using UPARSE (V7.0.1090). Each sequence was annotated by comparing the Ribosomal Database Project (RDP) classifier (V2.11)^1^ against the SILVA (SSU123) database^2^ using a comparison threshold of 70%. Alpha diversity was analyzed through three indices, namely Shannon, ACE, and Chao indices, and was calculated using mothur (V1.30.2)^3^. Principal coordinates analysis (PCoA) on weighted and unweighted UniFrac distance matrix was used for beta diversity analysis. The differences in the composition of gut microbiota among the three regions were detected by linear discriminant analysis effect size (LEfSe). PICRUSt 2 was used to predict the metabolic pathways of intestinal microbiota and investigate the functional differences in the microbial communities in samples from the three regions.

The R and SPSS (version 20.0) software packages were used for statistical analysis. Significant differences among groups were analyzed using one-way ANOVA, followed by Dunnett’s *post hoc* test, and *p* < 0.05 was defined as significant.

## Results

### Alpha Diversity of Microbial Communities in Yaks From Different Regions

**FIGURE 2:**
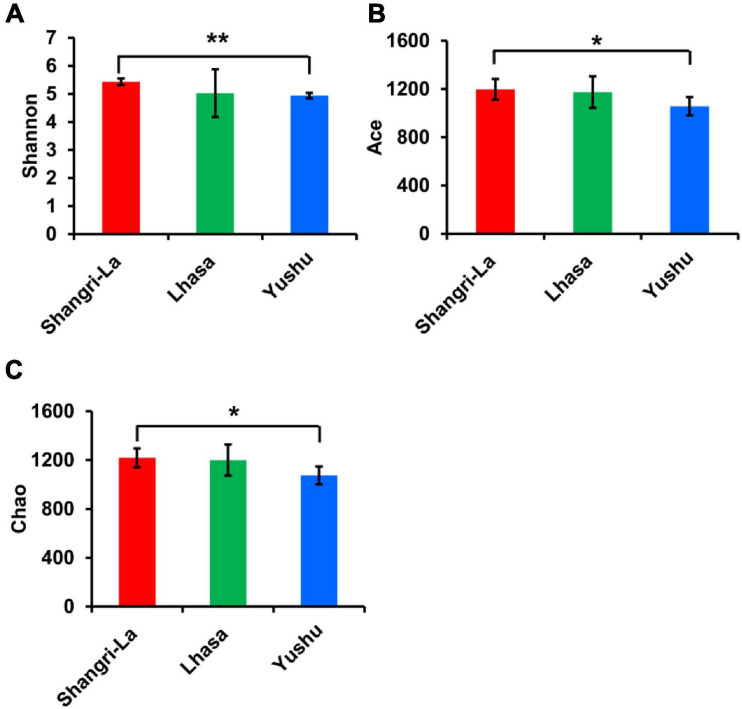
Alpha diversity of gut bacteria among different places. **(A)** The Shannon index of each region. **(B)** The ACE index of each region. **(C)** The Chao index of each region. *indicates that differences between groups are significant, *p* < 0.05; **indicates that differences between groups are very significant, *p* < 0.01.

A total of 160271 effective tags were generated from all the samples, including Lhasa 50507 reads, Shangri-la 52282 reads, and Yushu 57482 reads, with average read lengths of 436, 434, and 434 bp, respectively. Microbial diversity was analyzed using the Shannon diversity index, and richness was analyzed using Chao and ACE indices (Figure 2). The Shannon, ACE, and Chao indices were the highest in the Shangri-la samples (Figures 2A–C), suggesting that the diversity and richness of intestinal microbiota are the greatest for yaks from Shangri-la. The samples from Yushu had the lowest Shannon, ACE, and Chao indices. There were no significant differences in the Shannon, ACE, or Chao indices between the Lhasa and Yushu samples.

### Beta Diversity of Microbial Communities in Yaks From Different Regions

**FIGURE 3:**
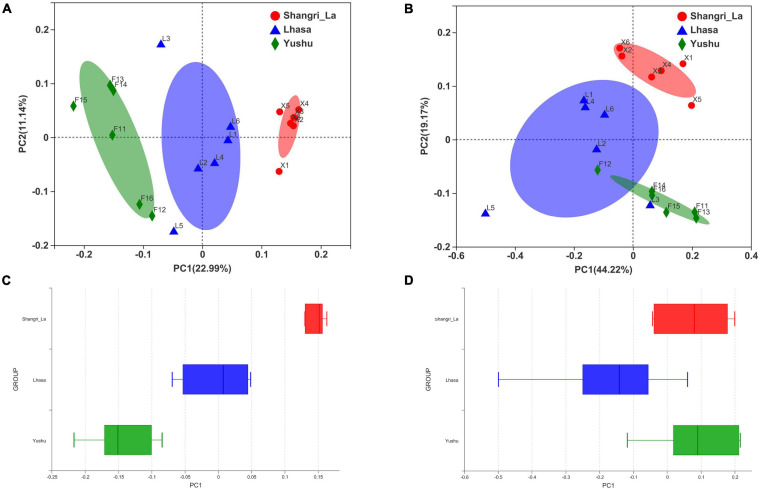
Principal coordinates analysis (PCoA) of gut microbiota between different regions. **(A)** PCoA results based on unweighted UniFrac distance of bacterial communities from different regions. **(B)** PCoA results based on weighted UniFrac distance of bacterial communities from different regions. **(C)** The distinct separation of samples from different regions shown on the PCo1 axis based on weighted UniFrac distance. **(D)** The distinct separation of samples from different regions shown on the PCo1 axis based on unweighted UniFrac distance.

Beta diversity was examined using PCoA. PCoA of the weighted (Figure 3A) and unweighted (Figure 3B) UniFrac distance matrices were carried out to reveal the differences in the bacterial community structure of the samples. The results of the PCoA showed distinct separation of samples from different regions based on the weighted UniFrac distance (Adonis: *R*^2^ = 0.4020, *p* = 0.001) and unweighted UniFrac distance (Adonis: *R*^2^ = 0.3026, *p* = 0.001). Furthermore, box plots of the first principal coordinate showed that samples from different regions were separated from each other (Figures 3C,D), revealing that yaks from each region host their own distinct gut microbiota.

### Composition of Gut Microbiota of Yaks From Different Regions

**FIGURE 4:**
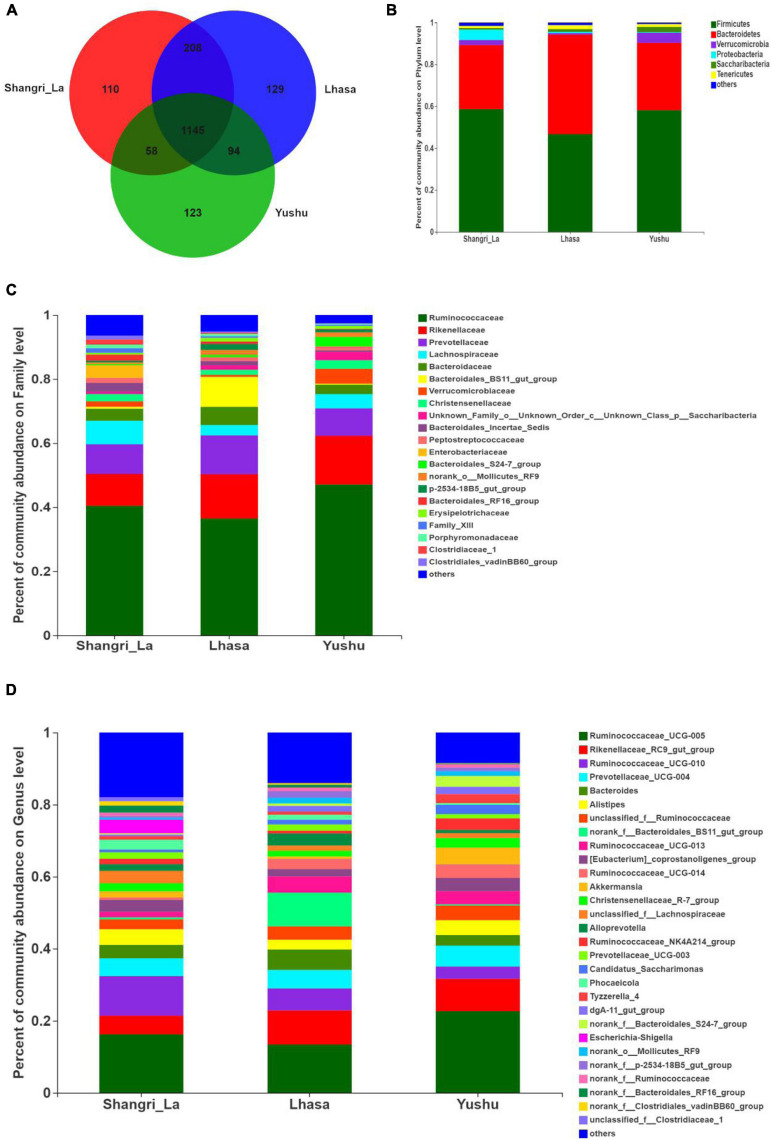
Composition of gut microbiota of yaks from different regions. **(A)** Venn analysis. **(B)** Relative abundance of community at the phylum level. **(C)** Relative abundance of community at the family level. **(D)** Relative abundance of community at the genus level.

Venn analysis (Figure 4A) showed that 1145 OTUs were shared among the three regions, indicating that Shangri-La, Lhasa, and Yushu had similar OTU distribution. However, some OTUs were unique to some regions; there were 110 unique OTUs in Shangri-la, 129 in Lhasa, and 123 in Yushu.

**FIGURE 5:**
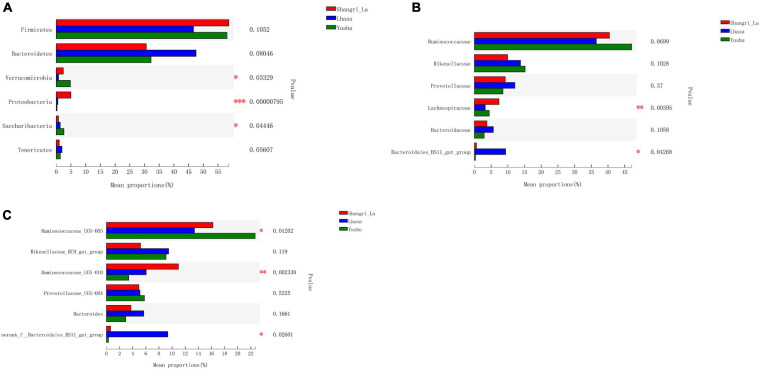
Statistical comparison of the relative abundance of yak gut bacteria among different regions. **(A)** Comparison of dominant phyla in the Shangri-la, Lhasa, and Yushu groups. **(B)** Comparison of dominant families in the Shangri-la, Lhasa, and Yushu groups. **(C)** Comparison of dominant genera in the Shangri-la, Lhasa, and Yushu groups.

We detected six phyla (Figure 4B and Supplementary Table 1), in the samples from the three regions. These phyla accounted for more than 0.2% of the community abundance at the phylum level. The dominant phyla were Bacteroidetes, Firmicutes, Verrucomicrobia, and Proteobacteria. Firmicutes and Bacteroidetes were the most represented phyla across all three regions, accounting for 89.26, 94.24, and 90.30% of the sequences in the Shangri-La, Lhasa, and Yushu groups, respectively. The relative abundance of Verrucomicrobia, Proteobacteria, and Saccharibacteria were significantly different among the samples from the three regions (Figure 5A).

We detected taxa from 21 families (Figure 4C) in the samples. The dominant families in Shangri-la included Ruminococcaceae, Rikenellaceae, Prevotellaceae, and Lachnospiraceae, with relative abundances of 40.43, 9.97, 9.25, and 7.35%, respectively. In Lhasa, the dominant families were Ruminococcaceae (36.50%), Rikenellaceae (13.81%), Prevotellaceae (12.12%), Bacteroidales_BS11_gut_group (9.34%), and Bacteroidaceae (5.67%). In Yushu, the major families included Ruminococcaceae (47.11%), Rikenellaceae (15.19%), and Prevotellaceae (8.56%). The relative abundance of Lachnospiraceae and Bacteroidales_BS11_gut_group was significantly different among all the samples (Figure 5B).

We detected 29 genera (Figure 4D) in the samples. Of these, *Ruminococcaceae _UCG-005* (16.23%), *Rikenellaceae_RC9_gut_group* (5.21%), and *Ruminococcaceae _UCG-010* (10.96%) were the dominant genera in the Shangri-la samples. In the Lhasa samples, *Ruminococcaceae_UCG-005* (13.39%), *Rikenellaceae_RC9_gut_group* (9.48%), *norank _f__Bacteroidales_BS11_gut_group* (9.34%), *Ruminococcaceae _UCG-010* (6.09%), *Bacteroides* (5.67), and *Prevotellaceae_UCG-004* (5.15%) were dominant. In the Yushu samples, *Ruminococcaceae_UCG-005* (22.65%) was the most dominant genus, followed by *Rikenellaceae_RC9_gut_group* (9.00%) and *Prevotellaceae_UCG-004* (5.80%). The relative abundance of *Ruminococcaceae_UCG-005*, *norank _f__Bacteroidales_BS11_gut_group*, and *Ruminococcaceae_UCG-010* exhibited significant differences among the different sample regions (Figure 5C).

**FIGURE 6:**
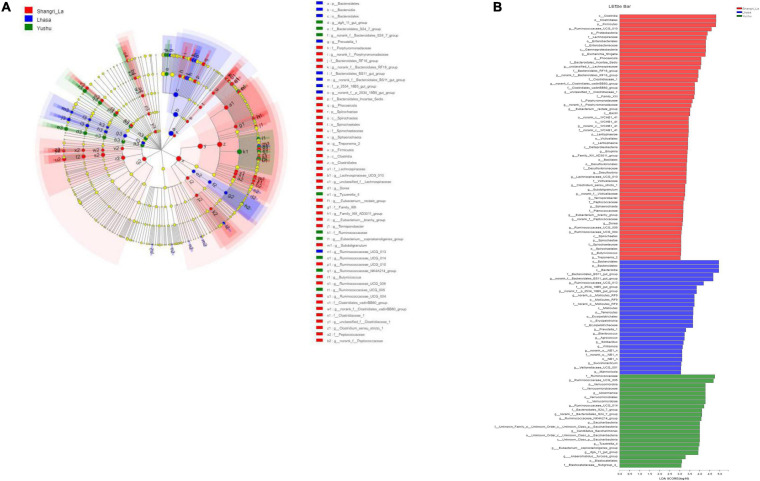
LEfSe analysis of gut microbiota. **(A)** Linear discriminant analysis (LDA) effect size (LEfSe) conducted on the intestinal microbiota of yaks from different regions (*p* < 0.05, LDA > 3). Different colors and sizes of nodes indicate microbial groups with significant differences and enriched species abundance. Light yellow nodes indicate groups with no significant difference. **(B)** LEfSe for three regions.

Linear discriminant analysis effect size revealed the relative abundance of the different bacterial taxa (from phylum to genus) in the samples from different regions (Figure 6A). Species with LDA scores >3 were considered as biological markers of the different groups (Figure 6B). The LEfSe results showed that 107 bacterial taxa were significantly enriched in the samples – 58, 27, and 22 enriched taxa were found in the Shangri-La, Lhasa, and Yushu samples, respectively. Signature gut microbes included Firmicutes, Clostridia, and *Ruminococcaceae_UCG-010* in the Shangri-La samples, Bacteroidetes in the Lhasa samples, and Ruminococcaceae in the Yushu samples.

### Predicted Function and Metabolism of Gut Microbiota

PICRUSt 2 prediction of the metabolic pathways of gut microbiota based on 16S rRNA sequencing data revealed that pathways in metabolism had the highest relative abundance, accounting for 67.45, 68.32, and 67.81% of the identified pathways in the Shangri-La, Lhasa, and Yushu samples, respectively (Table 1 and Supplementary Table 2). A total of 12 biochemical pathways were identified among metabolic functions (Table 2). Functions in carbohydrate metabolism, global and overview maps, and amino acid metabolism were enriched in all samples. Functions in amino acid metabolism were significantly higher in Yushu samples than in samples from the other regions (*p* < 0.05).

**TABLE 1:** The predicted functional composition (%) of the microbiota of yaks from different regions.

**Functions**	**Shangri-La (*n* = 6)**	**Lhasa (*n* = 6)**	**Yushu (*n* = 6)**
Cellular processes	6.07 ± 0.19^a^	5.63 ± 0.34^b^	5.98 ± 0.22^a^
Environmental information processing	7.14 ± 0.29^a^	6.19 ± 0.52^b^	6.58 ± 0.32^b^
Genetic information processing	12.86 ± 0.09^b^	13.31 ± 0.57^a^	13.18 ± 0.14^ab^
Human diseases	4.17 ± 0.03^a^	4.20 ± 0.05^a^	4.15 ± 0.09^a^
Metabolism	67.45 ± 0.52^b^	68.32 ± 0.38^a^	67.81 ± 0.56^ab^
Organismal systems	2.31 ± 0.03^a^	2.35 ± 0.08^a^	2.29 ± 0.03^a^

*Letters^(a,b)^ indicate significant differences within the same row (*p* < 0.05).*

**TABLE 2:** Predicted functional composition (%) of microbiota relating to metabolism.

**Metabolic pathway**	**Shangri-La (*n* = 6)**	**Lhasa (*n* = 6)**	**Yushu (*n* = 6)**
Amino acid metabolism	10.41 ± 0.05^*c*^	10.58 ± 0.16^b^	10.75 ± 0.07^a^
Biosynthesis of other secondary metabolites	1.94 ± 0.05^b^	2.02 ± 0.05^a^	1.97 ± 0.06^ab^
Carbohydrate metabolism	14.27 ± 0.21^a^	14.15 ± 0.10^ab^	13.99 ± 0.13^b^
Energy metabolism	6.40 ± 0.07^b^	6.54 ± 0.13^a^	6.39 ± 0.07^b^
Global and overview maps	12.84 ± 0.14^b^	12.79 ± 0.41^b^	13.20 ± 0.09^a^
Glycan biosynthesis and metabolism	2.37 ± 0.21^a^	2.63 ± 0.34^a^	2.37 ± 0.19^a^
Lipid metabolism	2.86 ± 0.02^a^	2.92 ± 0.07^a^	2.86 ± 0.06^a^
Metabolism of cofactors and vitamins	6.17 ± 0.07^a^	6.20 ± 0.09^a^	6.15 ± 0.10^a^
Metabolism of other amino acids	1.79 ± 0.03^ab^	1.85 ± 0.11^a^	1.76 ± 0.03^b^
Metabolism of terpenoids and polyketides	1.47 ± 0.01^b^	1.55 ± 0.09^a^	1.50 ± 0.02^ab^
Nucleotide metabolism	5.89 ± 0.05^ab^	6.06 ± 0.25^a^	5.85 ± 0.02^b^
Xenobiotics biodegradation and metabolism	1.06 ± 0.01^a^	1.03 ± 0.07^a^	1.01 ± 0.02^a^

*Letters^(a,b,c)^ indicate significant differences within the same row (*p* < 0.05).*

Other functional roles in the microflora of the samples included cellular processes, organismal systems, environmental information processing, human diseases, and genetic information processing (Table 3 and Supplementary Table 3). Additionally, pathways involved in replication and repair and translation were predominant in samples from each region. There were significant differences in the abundance of pathways related to infectious diseases: bacterial in the samples (*p* < 0.05). The functional roles in membrane transport in samples from Shangri-La were significantly higher than in those from the other regions (*p* < 0.05). Functional roles in folding, sorting and degradation, drug resistance: antineoplastic, and cancers: specific types in samples from Shangri-La were significantly lower than in those from the other regions (*p* < 0.05). The abundance of functional pathways for cell motility, cell growth and death, the digestive system, cellular community – prokaryotes, environmental adaptation, signal transduction, the nervous system, and aging in the samples from Lhasa were significantly different from those in samples from the other regions (*p* < 0.05). Human diseases from the immune diseases and endocrine and metabolic diseases pathways were significantly different in samples from Yushu than in those from other regions (*p* < 0.05).

**TABLE 3:** Percentage composition of other functional genes of the microbiota of yaks from different regions.

**Functions**	**Shangri-La (*n* = 6)**	**Lhasa (*n* = 6)**	**Yushu (*n* = 6)**
**Cellular processes**			
Cell motility	1.58 ± 0.15^a^	1.26 ± 0.26^b^	1.56 ± 0.18^a^
Cell growth and death	0.93 ± 0.03^b^	1.01 ± 0.06^a^	0.94 ± 0.04^b^
Transport and catabolism	0.35 ± 0.05^a^	0.41 ± 0.07^a^	0.35 ± 0.05^a^
Cellular community – eukaryotes	0.00 ± 0.00^a^	0.00 ± 0.00^a^	0.00 ± 0.00^a^
Cellular community – prokaryotes	3.21 ± 0.12^a^	2.92 ± 0.21^b^	3.13 ± 0.14^a^
**Environmental information processing**			
Signal transduction	2.83 ± 0.11^a^	2.58 ± 0.14^b^	2.75 ± 0.10^a^
Membrane transport	4.30 ± 0.18^a^	3.61 ± 0.39^b^	3.84 ± 0.22^b^
Signaling molecules and interaction	0.00 ± 0.00^a^	0.00 ± 0.00^a^	0.00 ± 0.00^a^
**Genetic information processing**			
Replication and repair	4.76 ± 0.02^b^	4.96 ± 0.25^a^	4.87 ± 0.04^ab^
Translation	5.49 ± 0.06^b^	5.71 ± 0.26^a^	5.64 ± 0.07^ab^
Transcription	0.26 ± 0.00^a^	0.26 ± 0.01^a^	0.26 ± 0.01^a^
Folding, sorting and degradation	2.34 ± 0.02^b^	2.39 ± 0.07^a^	2.40 ± 0.02^a^
**Human diseases**			
Cancers: Overview	0.64 ± 0.01^a^	0.66 ± 0.03^a^	0.65 ± 0.02^a^
Cardiovascular diseases	0.27 ± 0.00^ab^	0.26 ± 0.02^b^	0.28 ± 0.00^a^
Immune diseases	0.05 ± 0.00^a^	0.05 ± 0.01^a^	0.04 ± 0.00^b^
Infectious diseases: Parasitic	0.03 ± 0.00^b^	0.05 ± 0.03^a^	0.04 ± 0.01^ab^
Drug resistance: Antimicrobial	1.55 ± 0.02^a^	1.48 ± 0.07^b^	1.49 ± 0.02^ab^
Infectious diseases: Bacterial	0.70 ± 0.01^a^	0.67 ± 0.03^b^	0.64 ± 0.00^*c*^
Drug resistance: Antineoplastic	0.36 ± 0.00^b^	0.38 ± 0.01^a^	0.39 ± 0.01^a^
Endocrine and metabolic diseases	0.33 ± 0.00^b^	0.32 ± 0.01^b^	0.34 ± 0.00^a^
Neurodegenerative Diseases	0.15 ± 0.01^b^	0.18 ± 0.02^a^	0.16 ± 0.02^ab^
Cancers: Specific types	0.09 ± 0.00^b^	0.11 ± 0.01^a^	0.10 ± 0.01^a^
Infectious diseases: Viral	0.01 ± 0.00^a^	0.03 ± 0.01^a^	0.03 ± 0.01^a^
Substance dependence	0.00 ± 0.00^a^	0.00 ± 0.00^a^	0.00 ± 0.00^a^
**Organismal systems**			
Circulatory system	0.00 ± 0.00^a^	0.00 ± 0.00^a^	0.00 ± 0.00^a^
Environmental adaptation	0.21 ± 0.00^a^	0.20 ± 0.01^b^	0.20 ± 0.00^a^
Endocrine system	0.86 ± 0.01^a^	0.89 ± 0.04^a^	0.86 ± 0.01^a^
Digestive system	0.05 ± 0.01^b^	0.07 ± 0.02^a^	0.05 ± 0.01^b^
Excretory system	0.04 ± 0.00^ab^	0.03 ± 0.01^b^	0.05 ± 0.00^a^
Immune system	0.44 ± 0.01^a^	0.43 ± 0.01^a^	0.44 ± 0.01^a^
Nervous system	0.29 ± 0.01^a^	0.27 ± 0.01^b^	0.29 ± 0.01^a^
Aging	0.42 ± 0.01^b^	0.45 ± 0.03^a^	0.40 ± 0.01^b^
Development	0.00 ± 0.00^a^	0.00 ± 0.00^a^	0.00 ± 0.00^a^

*Letters^(a,b,c)^ indicate significant differences within the same row (*p* < 0.05).*

## Discussion

Alpha diversity is the measure of species diversity in an area and is represented by the Chao, Shannon, and ACE indices. Beta diversity represents the regional differences in species composition, which can provide valuable information about how the microbiota differ (Wang et al., 2020). To better understand the diversity in gut microbiota of yaks from different regions, we analyzed our data using the Shannon, ACE, and Chao indices as well as PCoA. The Shannon, ACE, and Chao indices revealed significant differences in the diversity of gut microbiota between samples from Shangri-la and Yushu. The samples from Shangri-la had higher Shannon, ACE, and Chao indices than those from Lhasa and Yushu. Additionally, analysis of beta diversity revealed a distinct separation between samples from different regions. This may be due to increasing environmental radiation from temperate regions to high-altitude cold regions, which is accompanied by a shift in the vegetation that may cause regional variations in the diet of yaks. It is also probable that the high ultraviolet levels, low temperature, and low oxygen levels in the Qinghai-Tibetan Plateau habitats affect the diversity of yak bacterial communities (Li and Zhao, 2015). In conclusion, the diversity of the intestinal microbiota of yaks is closely related to geographical location and conditions.

Venn diagrams have often been used to determine the number of common and unique microbial species in samples, and can intuitively represent similarities and overlaps in species from different environments (Shade and Handelsman, 2012). In this study, Venn analysis and relevant abundance analysis revealed that core microbiota, as well as a quantity of unique microbiota, were present in the samples from each region; only some bacteria were significantly different between samples from different regions. A total of six phyla, 21 families, and 29 genera were detected in the samples from the three regions. We found that Firmicutes and Bacteroidetes were the dominant phyla in all the samples. In previous studies, these two phyla were found to be dominant in the gut microbiota of yaks (Liu et al., 2019; Ma et al., 2019), pikas (Li et al., 2019), goats (Lei et al., 2018), and sheep (Cui et al., 2019), indicating that they may be essential components of the mammalian intestinal microbiota (Fan et al., 2020). Firmicutes and Bacteroidetes are important for the digestion of proteins and carbohydrates (Spence et al., 2006). Our results suggest that the high abundance of Firmicutes and Bacteroidetes found in yaks is possibly related to high energy consumption in cold environments (Zhang L. et al., 2020). We also found that the abundance of Verrucomicrobia, Proteobacteria, and Saccharibacteria were significantly different in the microbiota of yaks from different regions, which may be related to host diet and physiology (Kurilshikov et al., 2017). Studies have revealed that a lower abundance of Verrucomicrobia is correlated with increased fiber intake (Li et al., 2016). Yaks from Lhasa have a higher fiber intake than those from the other two regions. Ruminococcaceae, which is known to play an important role in cellulose and starch degradation (Jindou et al., 2006; Ze et al., 2012), was the most dominant family found in the microbiota of yaks from all three regions. The relative abundance of Ruminococcaceae was higher in the Yushu samples than in the other samples. This may be due to a high lignin and cellulose content in the diet of yaks from Yushu. The genus *Ruminococcaceae_UCG-005* belongs to the Ruminococcaceae family and is also important for cellulose digestion. It was predominant in all three regions. Therefore, the composition of the yak gut microbiome depends on the geographical region and corresponding conditions.

Gut microbiota have an important effect on the host’s immune development, absorption, degradation of nutrients, and enzyme metabolism (Martin et al., 2010). Hence, it is essential to better understand the mechanisms that adapt the gut to different environments. Our PICRUSt2 analysis revealed that the functional gene composition of yak intestinal microbes was similar in yaks from the three regions; similar results were found for the bifidobacterial community of Chinese human subjects from different regions (Yang et al., 2020). Microbial communities often show incredible taxonomic diversity, however, their functional compositions are not directly related to taxonomic diversity because some similar gene functions are encoded by many concomitant but taxonomically distinct microbes (Louca et al., 2018). We found that the functional gene composition of the gut microbiota of yaks from different geographical regions was similar.

## Conclusion

This study is the first to analyze the intestinal microbiota of yaks from three different geographical regions in China using high-throughput sequencing. The results indicate that the diversity and composition of the yak gut microbiota are related to the geographical regions. However, there were no significant differences in the functional gene composition of the microbiota of yaks from the different regions. Our findings provide a basis for studying the association between the intestinal flora of yaks and their adaptation to different geographical environments. Our study also lays the foundation for further exploring probiotic resources in the Qinghai-Tibetan Plateau.

## Data Availability Statement

The datasets presented in this study can be found in online repositories. The names of the repository/repositories and accession number(s) can be found below: NCBI (accession: PRJNA717166).

## Author Contributions

HS: conceptualization. WL: methodology. JX, SZ, YL, and YY: resources. QW: writing – original draft preparation. JS: writing – review and editing. JX, HS, and PX: supervision, project administration, and funding acquisition. All authors have read and agreed to the published version of the manuscript.

## Conflict of Interest

The authors declare that the research was conducted in the absence of any commercial or financial relationships that could be construed as a potential conflict of interest.

## Footnotes

1 https://sourceforge.net/projects/rdp-classifier/

2 https://www.arb-silva.de/

3 https://www.mothur.org/wiki/Download_mothur
